# Atypical Presentation of Hirayama Disease Involving the Cervico-Thoracic Segment Causing Diagnostic Dilemma: A Case Report

**DOI:** 10.7759/cureus.34396

**Published:** 2023-01-30

**Authors:** Sanket Mishra, Deepankar Satapathy, Nego Zion, Udeepto lodh

**Affiliations:** 1 Department of Orthopaedic Surgery, Institute of Medical Sciences (IMS) and SUM Hospital, Bhubaneswar, IND

**Keywords:** monomelic amyotrophy, juvenile muscular atrophy, thoracic spine, lower motor, hirayama disease

## Abstract

Hirayama disease, also known as monomelic amyotrophy, usually affects young males who initially experience increasing muscle weakness and atrophy of the distal upper limb before experiencing a sudden plateauing of symptom progression a few years later. It is a form of cervical myelopathy characterized by self-limiting, asymmetrical lower motor weakness of the upper limbs affecting the hands and forearms. This condition is brought on by the cervical dural sac and spinal cord being abnormally displaced forward during neck flexion, which causes the anterior horn cells to atrophy. However, research into the precise process is ongoing. Patients presenting with such features with additional atypical symptoms, like back pain, weakness, atrophy and paresthesia of lower extremities cause a diagnostic dilemma. We describe a case of a male patient, age 21, who complained of weakness in both upper limbs mostly on the hand and forearm muscles along with weakness and deformity in both lower limbs. He was diagnosed with atypical Cervico-thoracic Hirayama disease and treated.

## Introduction

Hirayama disease is an uncommon disorder marked by selective muscular atrophy with unilateral or bilateral asymmetrical regional muscular weakness of upper limb usually involving C7, C8, and T1 nerve roots. It was initially reported and described by Keizo Hirayama in 1959 who described it as “juvenile muscular atrophy of unilateral upper extremity” [1]. To further define the condition, Gourie-Devi et al. established the name "monomelic amyotrophy" in 1984 [2]. In 1989, the first pathological research was described [3]. It was thought to be a type of motor neuron disorder that resulted in the atrophy of the affected upper extremity muscles as a result of growth imbalances between the spinal canal's contents and the vertebral column, which led to an unequal thickening of the dural sac. The posterior dural sac moves anteriorly during neck flexion as a result of these growth imbalances, which dynamically compresses the anterior portion of the cervical cord. Relatively young males in the age group of 15 and 25 years are most frequently affected (20:1 male-to-female ratio) and it typically manifests as a slow beginning and benign course [4].

Classical Hirayama is characterised by slow, progressive muscle atrophy of the upper limb(s) that often reaches a plateau over a number of years before plateauing; and in some cases, mild to moderate recovery. Rarely are sensory and autonomic functions involved [5]. Additionally, lower limb involvement, which is usually rare, can make a diagnosis more difficult. Cervical MRI abnormalities that are indicative of Hirayama disease include increased posterior epidural space with prominent venous plexus, lower cervical spinal cord atrophic changes, hyperintense signal on T1 weighted images, loss of the dural attachment to the lamina with forward displacement, and reduced cervical lordosis [6,7]. Its prevalence in Asian countries is considered higher. Over 35 years period about 279 cases have been reported from India itself [8].

We report one such case, with typical MRI features but atypical spinal segment involvement and clinical features of Hirayama disease.

## Case presentation

A 21-year-old male patient presented with progressive weakness and atrophy of both upper and lower limbs of 8-10-year duration. Symptoms first appeared in the upper limbs at the age of 14 years, as asymmetrical weakness of both upper limbs. The weakness did not affect daily activities so the patient neglected the symptoms till relatives described thinning of both upper limbs and loss of muscle bulk. The patient also had a history of occasional tingling in both of his hands which was left unattended. The local hospital could not detect any ailment and the patient was discharged with few medications. Over the next few years, the patient started having difficulty in gripping and holding objects. He found it difficult to ride a bicycle and write. In addition, he started feeling clumsiness of both lower limbs and found it difficult to run and play football. There were no bladder and bowel abnormalities. He was taken to the nearest medical college, where suspicion of motor neuron disease was made by preliminary investigations. Chest and cervico-dorsal plain radiographs, cervical MRI, brain CT and MRI, and infection screening including TB were all negative, so no conclusive diagnosis was made. The patient’s weakness and atrophy involved both upper and lower limbs. The symptoms continued to progress for the next two years and the patient was unable to lift heavy objects or ride a bicycle but he was able to walk unassisted. There was occasional back pain. By the age of 17 years, the patient started feeling better and was able to walk much better and ride a bicycle again. He attempted to play football, but his limbs continued to be very atrophic and thin and did not improve with time. Since the last two years from his 19th birthday onwards, the patient again noted worsening of symptoms and accelerated deformity and weakness of both lower limbs. Back pain has increased, both hands and forearm appear even more atrophic and the patient can barely walk or grip things at the time of presentation.

**Figure 1:**
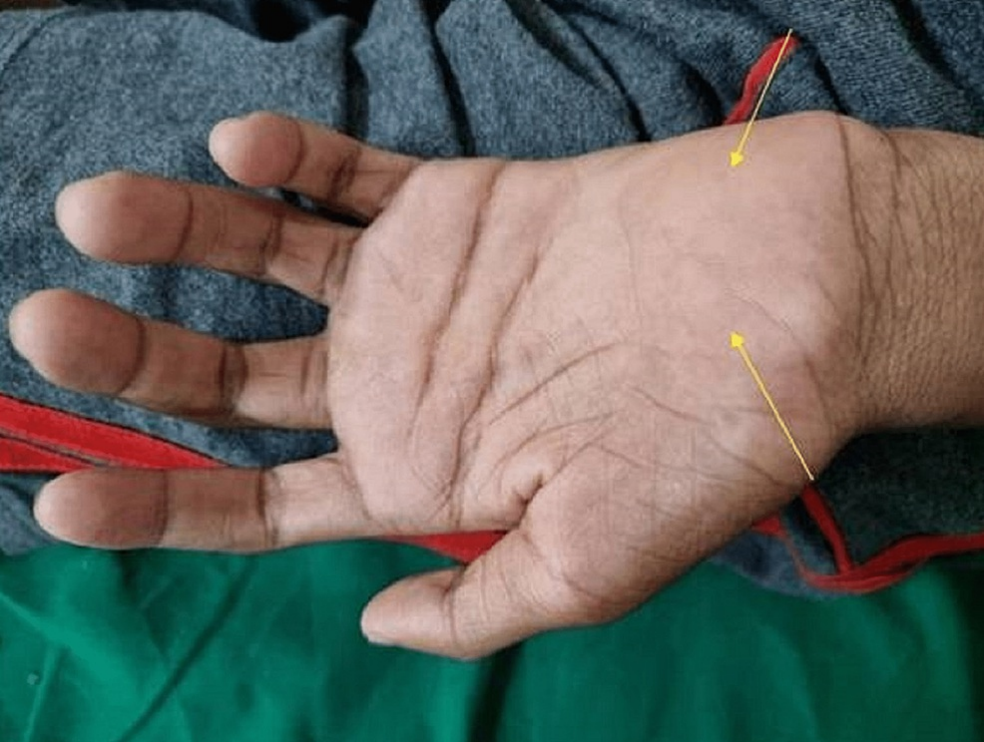
Prominent thenar and hypothenar atrophy of left hand (yellow arrows).

**Figure 2:**
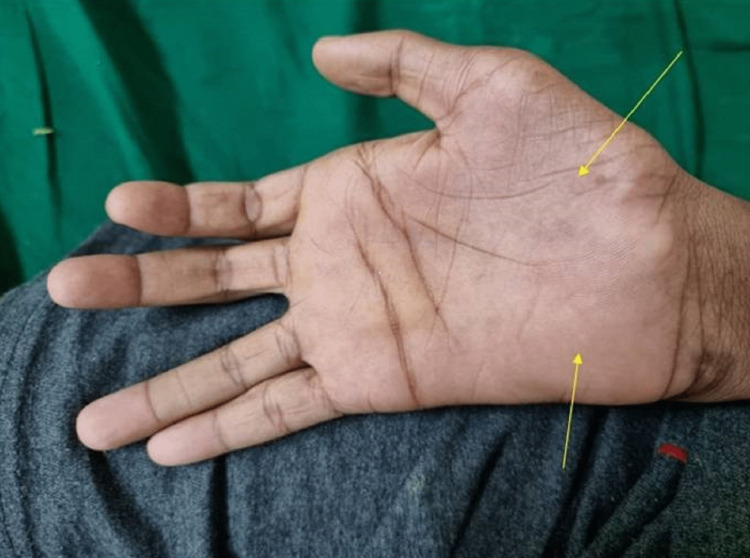
Prominent thenar and hypothenar atrophy of right hand (yellow arrows).

**Figure 3:**
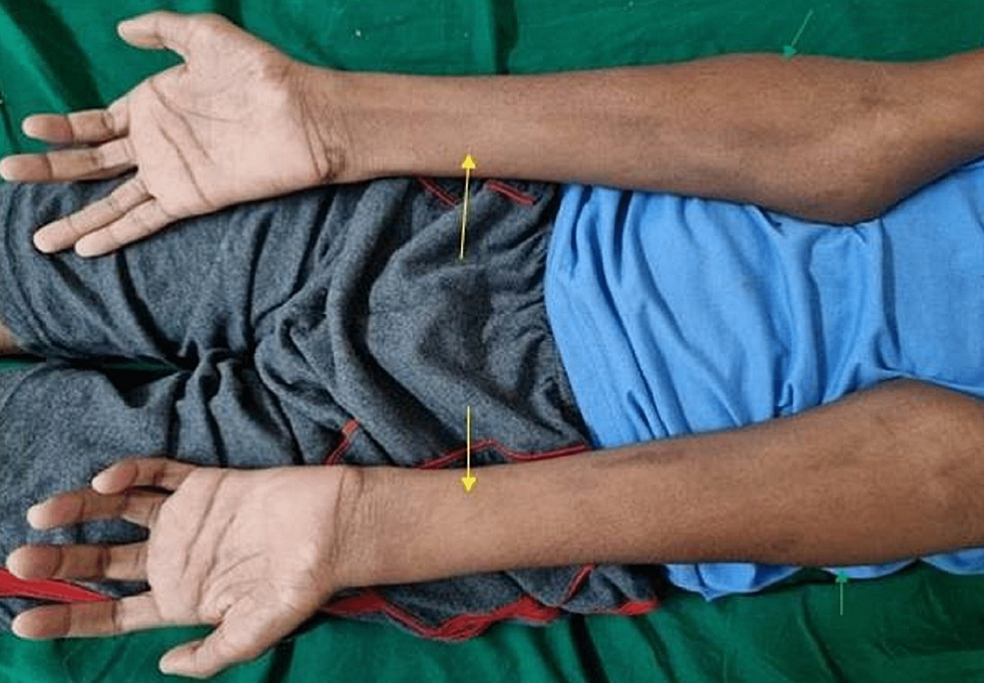
Prominent bilateral forearm muscle atrophy (yellow arrows), selective sparing of brachioradialis (green arrows).

The boy had normal speech and higher mental processes and neurological examination revealed an attentive, conscious and oriented individual. Higher cranial nerves examinations were within normal limits. Examination of the upper limbs revealed forearm, interosseous, thenar, and hypothenar muscular atrophy and weakness (Figures 1-3).

**Figure 4:**
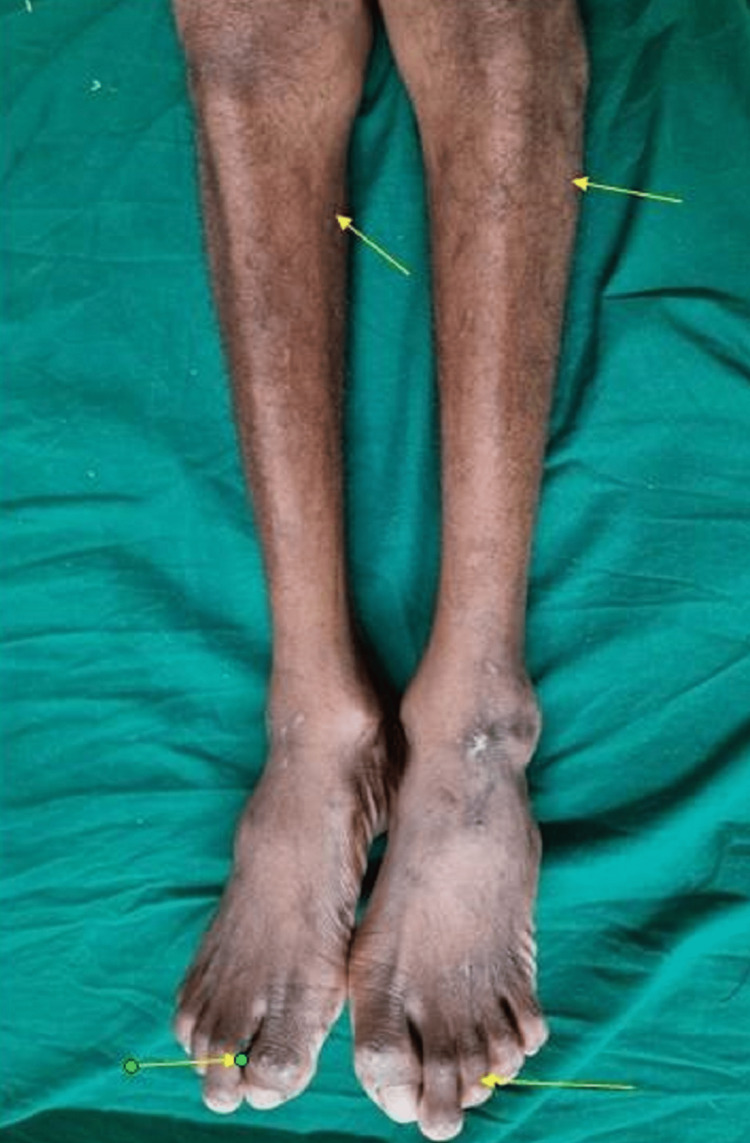
Atrophy of bilateral lower limb muscles, more marked in the calf muscles. Foot showing bilateral Hallux valgus deformity and clawing.

Examining the lower limbs indicated more extensive calf and thigh muscular atrophy. Clawing with the Hallux Valgus deformity was observed on both feet (Figure 4). Both the upper and lower limbs had normal deep tendon responses. Deep and superficial sensory examinations were normal. Gait and coordination were both typical. He didn't have any tremors, fasciculations, uncontrollable movements, or unusual perspiration. No bladder or bowel involvement was found. Respiratory rate was normal without any breathing difficulty. Electromyography (EMG) and nerve conduction velocity (NCV) test findings were suggestive of latency in the Median and Ulnar nerve conductions with no recording at the wrist for the right Median nerve. Similarly, there was latency seen in bilateral Posterior Tibial and Common Peroneal nerve conductions. Sensory studies were within normal limits. Such recordings were again suggestive of selective motor axon degenerative pathology, hence an early diagnosis of generalized motor neuron disease like amyotrophic lateral sclerosis (ALS) was made but lack of respiratory and abdominal muscle involvement and a brief period of spontaneous recovery and relative lesser involvement of other nerve roots made the diagnosis questionable.

**Figure 5:**
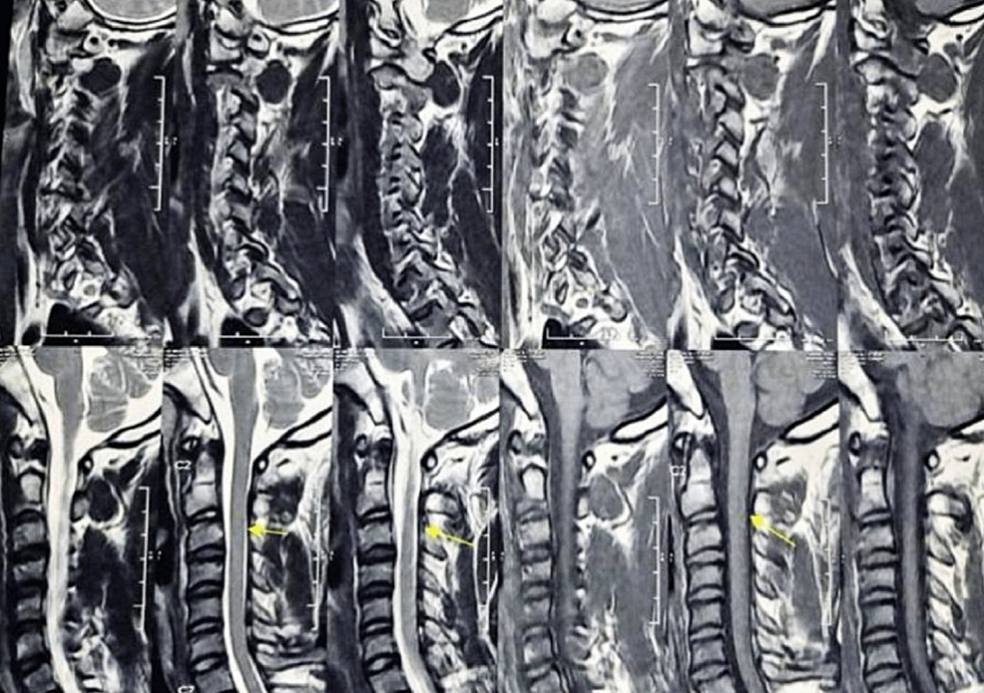
Normal appearing MRI sequence of the cervical region with a mild extension of the neck.

**Figure 6:**
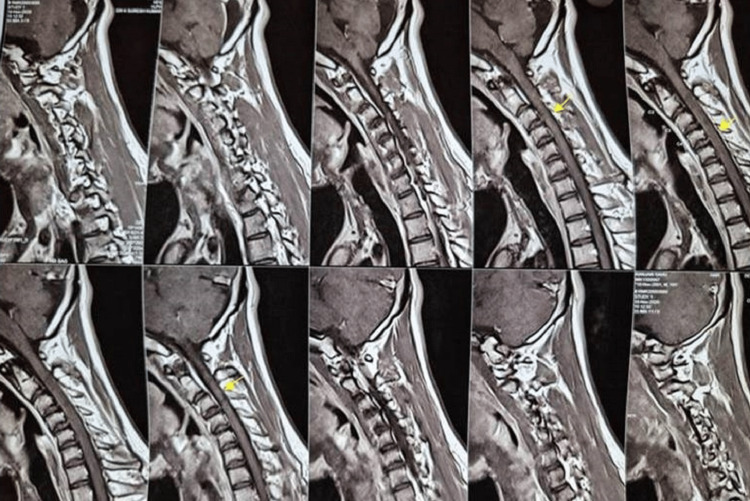
Normal appearing MRI sequence of the cervical region with neck flexion.

**Figure 7:**
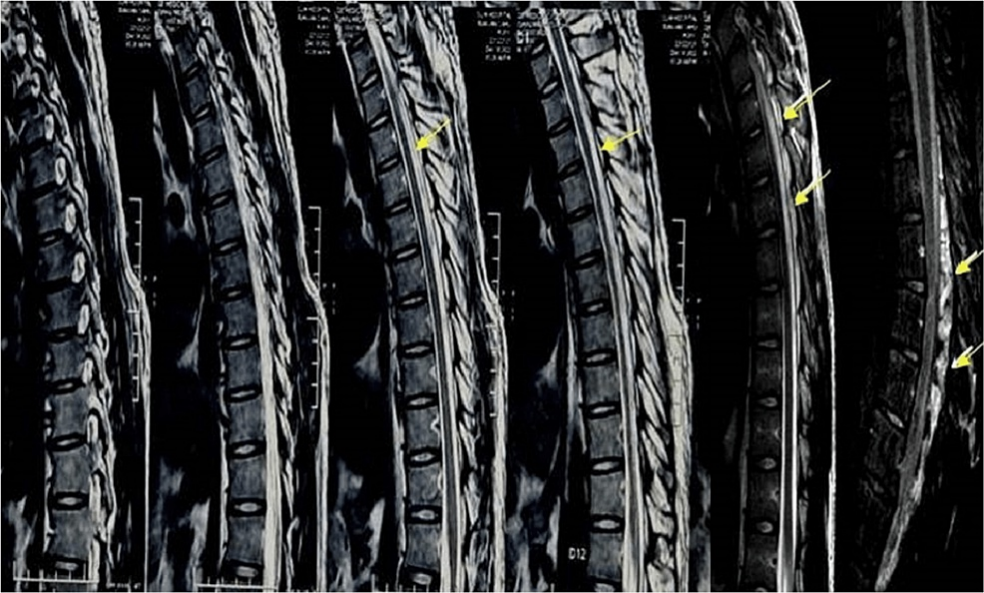
Flexion MRI sequence of thoracic spine showing increased posterior epidural space, further confirmed by contrast MRI showing increased venous prominence and posterior void (yellow arrows).

MRI of the brain was normal. MRI of the cervical spine with screening of other regions was normal (Figure 5 and Figure 6), but a cervico-thoracic flexion MRI revealed forward migration of the posterior wall of the dura mater indenting the spinal cord from C7-D7 levels with prominent posterior epidural space with fat and flow voids. On contrast MRI there was prominent homogenous posterior epidural enhancement (Figure 7). Routine blood test, rheumatoid profile, thyroid screening and Vit D levels were within normal levels.

Based on these findings a diagnosis of atypical Hirayama disease involving cervico-thoracic region was made. A neurosurgeon’s opinion was sought and duroplasty was advised. The patient refused surgery and is on regular follow-up. Although there has been no improvement in the patient's condition but there is also no further deterioration. The patient is at times able to walk without support. He is supplemented with neurovitamins like methylcobalamine, B1 and B6. The trial with steroids did not show any additional improvement so was discontinued. The patient continues physiotherapy and occupational rehabilitation and is relatively doing well.

## Discussion

Cervical C7, C8, and T1 segmental myotomes are commonly involved in Hirayama disease, a benign form of localised amyotrophy that spares the brachioradialis, which results in the recognisable oblique amyotrophy. There is also sparing of the proximal muscles innervated by C5-6 segments. With normal sensory and deep tendon reflex evaluation, it is characterised by asymmetrical weakness and wasting of the upper limb muscles that involves the aforementioned nerve roots. According to Hassan et al., the autonomic system is rarely implicated [5,9]. According to Tashiro et al. [10], the following criteria are important for diagnosis:

a. Forearm and hand weakness and atrophy with a distal predominance,

b. Single-sided affection of the upper extremity in the majority of cases,

c. Involving individuals in the age group of 10-20 years,

d. Uneventful and gradual onset with slow advancement for the initial few years, then later plateauing,

e. Non-involvement of the lower extremities,

f. Lack of sensory abnormality or normal deep tendon reflexes and

g. Screening and exclusion of other motor disorders.

Except point e, most of the other points were fulfilled by our case, which made diagnosis simpler. Hirayama disease's exact pathophysiology is still a mystery. Insufficient circulatory changes in the lower cervical cord is one of the primary causes, according to theories. The fundamental pathogenic mechanism of Hirayama illness is believed to be the anterior displacement of the posterior dural wall involving the lower cervical region with a flexed neck. This frequently causes the lower cervical cord to flatten asymmetrically [11,12]. Such displacement and loss of cervical dural attachment may be 100% specific and have a sensitivity of 70-93% for Hirayama [6,7]. In a classical case of Hirayama disease, there is increased length of the posterior wall during flexion which is caused by an imbalance in the growth of the vertebrae and dura mater, which results in a constricted dural canal and an overextended cord. The cord is subsequently compressed as a result of the posterior dural wall's anterior displacement. The anterior section of the spinal cord may experience chronic microcirculatory problems due to this compression, which could lead to necrosis of the anterior horns, which are most susceptible to ischemia [13]. In our cases, detachment of the dura over several thoracic levels was seen which is atypical to reported literature.

Asymmetric muscle weakness and visible atrophy usually involving the upper limbs are characteristic symptoms of patients with Hirayama disease who generally are young males [6]. Our patient was a young (age 21) male. The symptoms that were initially seen were unusual, including weakness and paresthesia in both upper and lower limbs, the shoulders, the lower back, and the lower legs and feet. The symptoms of the patient were typically worse in the lower extremities and aggravated by particular movements. In contrast to Hirayama disease's normal presentation of upper extremity weakness, atypical symptoms in this case were likely caused by the increased and aberrant ligamentous laxity in the upper and mid-thoracic spinal segments. Due to the self-limited nature of the condition with periods of spontaneous improvement and the relative lack of involvement of higher cranial nerves and the selective sparing of the respiratory, abdominal, and proximal extremity muscle groups, our case differs from traditional types of motor neuron diseases like amyotrophic lateral sclerosis and HIV-induced polyneuropathy, congenital neuropathies, post-polio syndrome and syringomyelia.

Monomelic amyotrophy is a self-limiting disorder without a cure. MRI and EMG/NCV are frequently required studies, along with clinical and laboratory diagnostic data for making diagnoses. Most of the treatment is conservative. Taylor’s Brace or cervical collars can be utilized to reduce symptom aggravation and minimize neck flexion and compression. Rarely is surgery necessary [14]. For cervical involvement, surgical procedures such as cervical fixation and posterior cervical decompression with epidural venous plexus coagulation are outlined, whereas for atypical thoracic involvement, duroplasty, decompression and/or conservative therapy constitute the main approaches.

## Conclusions

Hirayama disease is a rare form of motor neuron amyotrophy typically seen involving cervical spine causing asymmetrical upper limb weakness and atrophy with sparing of lower limb, without sensory deficit. Involvement of thoracic segment leads to atypical lower limb symptoms which have to be differentiated from other forms of motor neuron diseases and peripheral neuropathies based on thorough laboratory and radiological evaluation. Like in cervical, MRI is typical for diagnosing thoracic Hirayama. Treatment is still majorly conservative with splints and medication. Surgical procedures like posterior fixation and decompression and duroplasty are rarely indicated. The understanding about Hirayama disease is still evolving.
